# Case Report: Report of 2 Different Cases of Ovarian Teratoma Evaluated by Dynamic Contrast-Enhanced Ultrasound

**DOI:** 10.3389/fped.2021.681404

**Published:** 2021-06-11

**Authors:** Katja Glutig, Ilmi Alhussami, Paul-Christian Krüger, Matthias Waginger, Felicitas Eckoldt, Hans-Joachim Mentzel

**Affiliations:** ^1^Section for Pediatric Radiology, Institute for Diagnostic and Interventional Radiology, University Hospital Jena, Jena, Germany; ^2^Pediatric Surgery Clinic, University Hospital Jena, Jena, Germany

**Keywords:** teratoma, ovary, ultrasound, dynamic CEUS, pediatric

## Abstract

Ovarian masses are not easily differentiated on transabdominal ultrasound in children. A useful supplement in various pediatric applications is dynamic contrast-enhanced ultrasound (dynCEUS). It can be performed quickly and easily. However, the literature for dynCEUS on pediatric ovarian masses is limited. We compared two cases with ovarian teratoma in which dynCEUS was a helpful additional tool.

## Introduction

Non-specific chronic to acute abdominal pain is the primary clinical symptom of ovarian masses that often lead to a visit to the emergency department. Transabdominal B-mode ultrasound (US) is then the initial imaging modality. Neoplasms of the ovaries in girls are rare, with an incidence of only 3 cases per 100,000 per year (1), although true neoplasms are occasionally difficult to distinguish from other ovarian changes on B-mode ultrasound. This may be a hemorrhagic ovarian cyst, endometriosis, or torsion. Another differential diagnosis is a conglomerate tumor involving the ovary after a perforated appendicitis (2). In almost all cases, surgery is then the treatment of choice, although it should be performed sparing the ovary and preserving fertility (3). It is obligatory to assess the pathology of ovarian masses in advance with imaging and laboratory findings. Additional clinical clues include elevated tumor markers, virilization, and pre-mature puberty (4). Subsequently, the required surgical approach should be carefully planned accordingly (5).

Additional magnetic resonance imaging (MRI) is helpful in differentiation due to the advantageous high contrast resolution for soft tissue. Therefore, MRI is considered the gold standard of modern diagnostics without ionizing radiation in the evaluation of pelvic masses (6). Diffusion-weighted imaging (DWI) and the use of MRI contrast media in dynamic MRI also improve diagnostic accuracy. However, in younger children, sedation or anesthesia is often required for successful MRI examinations to achieve artifact-free imaging. However, MRI examinations under anesthesia or sedation are time-consuming, personnel-intensive, and costly.

Time-resolved dynamic contrast-enhanced ultrasound (dynCEUS) is another diagnostic modality. Lately, the use of dynCEUS has been increasing significantly in pediatric cases. Dynamic CEUS is quick and easy to perform in pediatric patients and is very well-tolerated (7). Visual analysis of microbubble contrast media (SonoVue^®^) distribution is possible during the examination. The intravenously applied microbubbles remain strictly intravascular and the gating of reflex microbubbles represents the vascularization and perfusion of a mass (8). Therefore, CEUS is well-suited to differentiate tumors, such as those of the ovaries. Several studies in adults describe the value of CEUS in differentiating benign from malignant ovarian tumors (9, 10). In contrast, the pediatric literature is limited (11). These two cases describe the findings on dynamic CEUS in pediatric patients and highlight the diagnostic differences in one case of a mature teratoma and another case of an immature teratoma with potential malignant changes. The aim of the work is to improve the understanding of dynamic CEUS in cases with of the ovary in children.

## Materials and Methods

### Ultrasound Protocol

Scans were performed with a Siemens (Sequoia, Siemens Healthineers, Erlangen, Germany) ultrasound machine by an experienced pediatric radiologist trained in CEUS with a 6-1 MHz curvilinear transducer in both cases using the same abdominal preset. In childhood and adolescence, the ultrasound contrast media is applied depending on age (0.1 ml/year of life) (7). Recently, the United States Food and Drug Administration (FDA) approved SonoVue^®^ for intravenous application in pediatric liver imaging. The recommended dosage depends on the weight of the child. The dosage is 0.03 ml per kg bodyweight with a maximum limit of 2.4 ml per injection (12). For contrast-enhanced ultrasound, our two cases received a standardized age-adapted amount of 0.1 ml SonoVue^®^ (Bracco Imaging, Germany) intravenously per year of life, followed by a 10 ml bolus of NaCl-solution.

One cycle of contrast was sufficient to characterize the lesion. For accurate quantitative evaluation of perfusion dynamics, a video clip of ~120 s was recorded and stored. For software evaluation, the position of the transducer was not allowed to change with the onset of contrast media injection.

**Figure 1:**
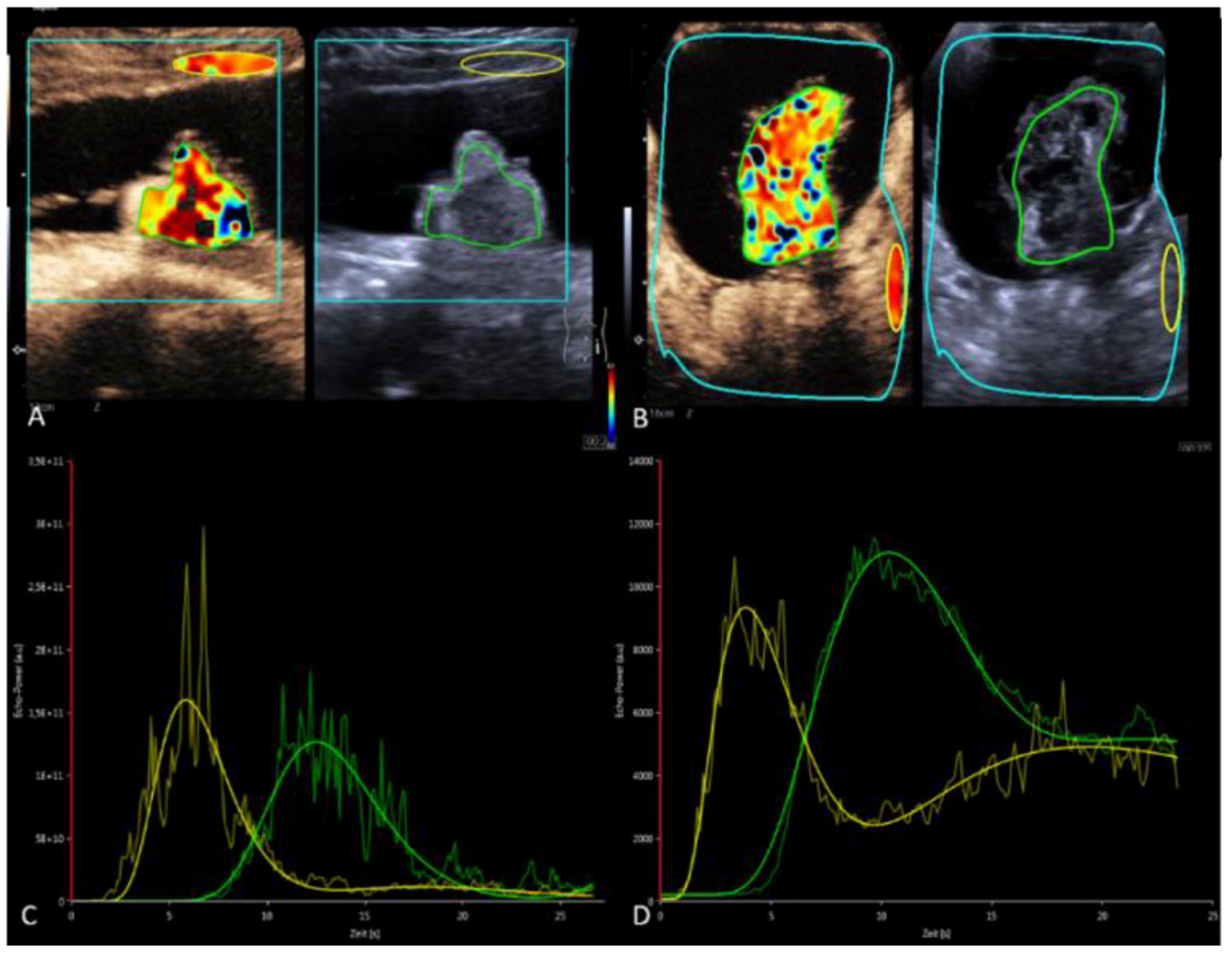
Quantitative analysis using VueBoxTM software (Bracco Imaging, Germany). **(A,B)** Case 1: mature teratoma. **(C,D)** Case 2 Immature teratoma with potential malignant changes. **(A,C)** CEUS with contrast image (left) and B-mode (right) in parallel mode. Color coding of RT within the set ROI's. Green ROI in the solid portion of the teratomas. Yellow reference ROI in subcutaneous fat **(A)** or retroperitoneal **(B)**. **(C,D)** Time-intensity curves—green curve: ROI measurement in the ovarian mass with visualization of PE, RT, TTP, AUC in case 1 and 2. Yellow curve: reference ROI.

For quantitative perfusion analysis of dynamic CEUS, a dedicated software (VueBox^®^, Bracco Imaging, Germany) was used (13). Time-intensity curves graphically represent the distribution of hyperreflective microbubbles in a lesion by placing a region of interest (ROI) in ellipsoidal or variable shape in the target lesion (Figure 1, green ROI) and another ROI in normal tissue (subcutaneous fat) as a reference (Figure 1, yellow ROI) (14). The evaluation software recorded parameters describing the enhancement, distribution, duration of enhancement, and washout of the bubbles. Peak enhancement (PE) as maximum signal intensity, time to peak (TTP) as the time to PE, rise time (RT), area under the curve at wash-in (WiAUC), and area under the curve at wash-in and wash-out (WiWoAUC) are the values with the strongest impact (15) (Table 1).

**Table 1:** Clinical characteristics, ultrasound data and dynamic CEUS parameters.

	**Case 1**	**Case 2**
	**Mature teratoma**	**Immature teratoma**
Age, y	16	10
Weight, kg	69	34
Height, cm	159	148
Body mass index, kg/m^2^	27.3	15.5
AFP (serum), ng/nl	2.1	15
Beta-HCG (serum), mlU/ml	1.2	<0.3
Amount SonoVue, ml	1.6	1.0
Ovary	left	left
Total, cm	15.4 × 11.6 × 5.3	15.5 × 9.5 × 12.3
Solid part, cm	4.2 × 3.0 × 3.2	7.0 × 4.2 × 6.3
PE relative, %	79	117
RT relative, %	162	223
TTP relative, %	124	267
WiAUC relative, %	126	259
WiWoAUC relative, %	119	255

*AFP, Alpha-Fetoprotein; Beta-HCG, human chorionic gonadotropin; PE, peak enhancement; RT, rise time; TTP, time to peak; WiAUC, wash in Area under the curve; WiWoAUC, wash in und wash out Area under the curve; cm, centimeter; y, year; kg, kilogram*.

Following the German Drug Law, the case series was approved by the local ethics committee under registration number 2021-2102. Written informed consent was obtained from all patients and their custodial parents.

## Cases

### Case 1: Mature Teratoma

A 16-year-old girl was presented to the emergency department with severe abdominal pain that was crampy for several minutes. Anamnesis revealed weight loss of 4 kg in 2 weeks and recurrent nausea, hypertensive attacks, and occasional menstrual pain. All laboratory tests, including inflammatory and tumor markers, were normal. Menarche was at the age of 13 years and regular menstruation since then.

**Figure 2:**
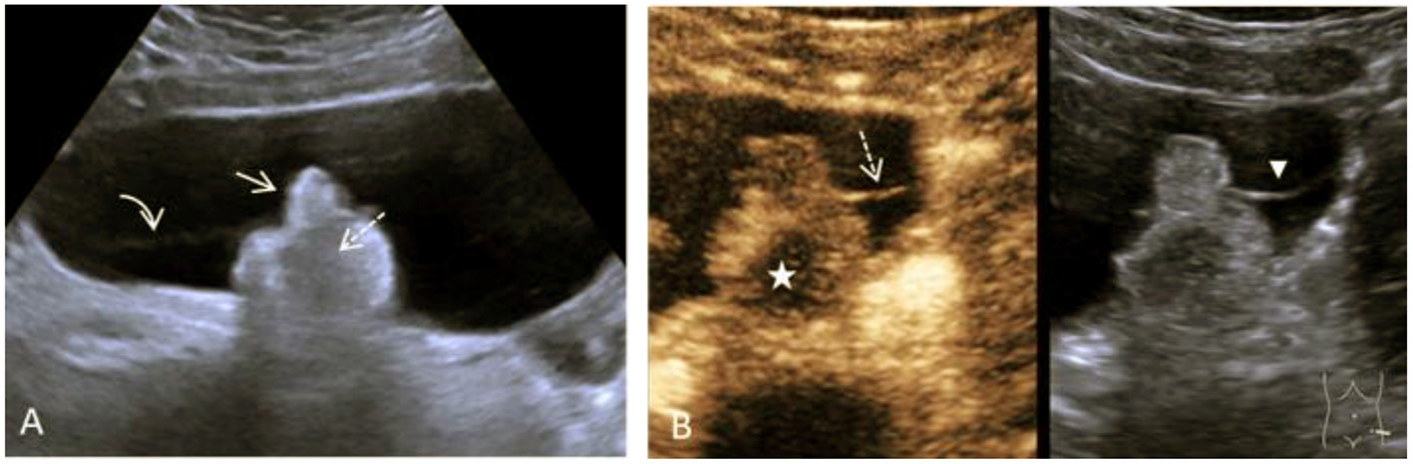
Case 1, a 16-year-old girl with a mature teratoma of the right ovary. **(A)**, B-mode Ultrasound showing a large cystic structure. In it, a solid lobulated portion, the so-called Rokitansky nodule, is adherent (arrow) to the dorsal wall. Within the solid echogenic mass, there are centrally echo-free areas with dorsal extinction (dashed arrow). There is very delicate septation between the solid part and the cyst wall (curved arrow). **(B)** CEUS of the solid part and the intracystic septation in the contrast image (left) and the native B-mode (right) in parallel mode. Image sequence ~35 s after application of the contrast media i.v.: The intracystic membrane is very delicately detectable in the native B-mode (arrowhead) and accumulates contrast bubbles (dashed arrow). The contrast media shows strong perfusion with numerous microbubbles in the left part of the solid parts, the central part is excluded (star). In conjunction with the histological findings of the mature teratoma, this is a bony tooth.

B-mode ultrasound and a multimodal MRI with DWI and contrast media showed a large cystic mass extending from the left ovary to 15.4 cm. Within the thin-walled cyst were a hyperechoic solid structure of about 4.2 cm and a delicate septum (Figure 2). Doppler ultrasonography showed non-specific perfusion.

CEUS examination visually verified early arterial accumulation of vesicles in the solid structure. A small central portion of the solid structure remained free of microbubble contrast media throughout the examination. In the quantitative analysis TTP in the solid part was 12.5 s, RT was 4.6 s, and relative PE was 79% (Figures 1A,C).

Based on the findings of multimodal MRI and dynamic CEUS, a diagnosis of mature teratoma was made, which was confirmed by laparoscopic surgery with unilateral tumor extirpation and histological workup. It was a mature teratoma without signs of malignancy.

### Case 2: Immature Teratoma With Potential Malignant Changes

**Figure 3:**
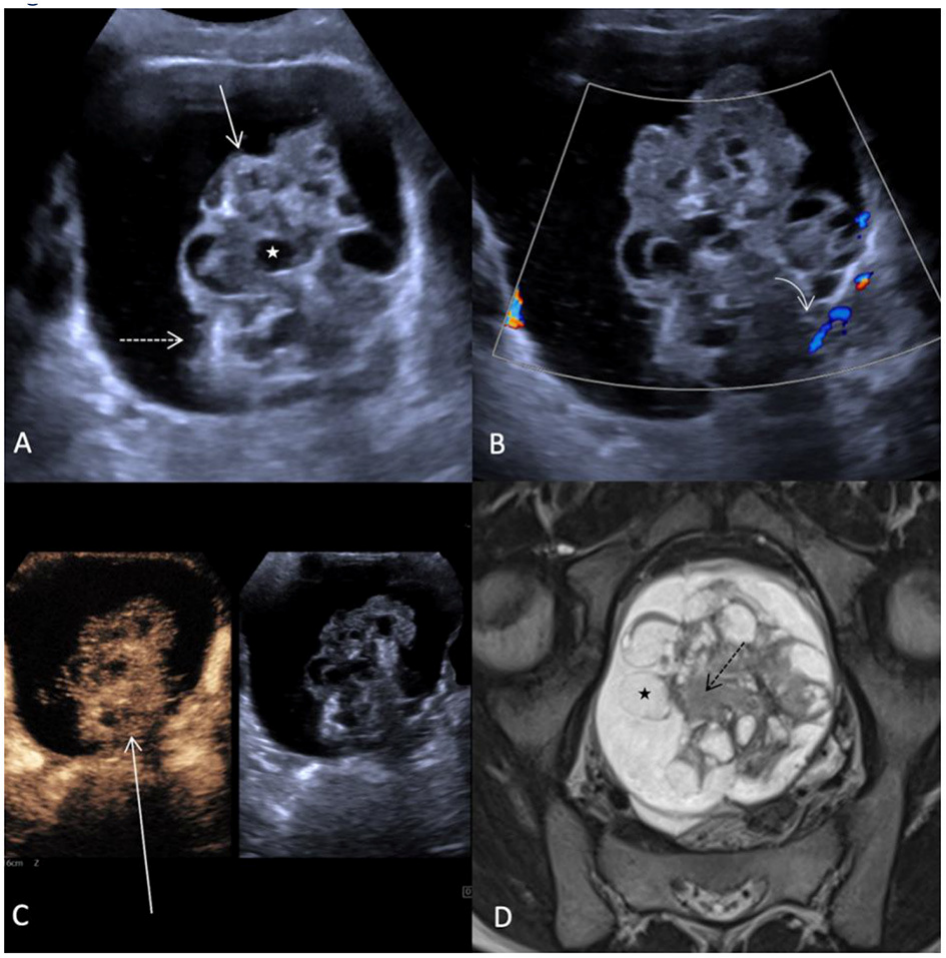
Case 2, 10-year-old girl with an immature teratoma with potential malignant changes. **(A)** B-mode ultrasound showing a cystic structure extending from the left ovary up to 16 cm in diameter. Within the cyst there is a solid part (arrow) measuring up to 7.5 cm. This is interspersed with an irregular surface (dashed arrow), inhomogeneous structure with multiple small cystoid lesions (white star). **(B)** In Doppler ultrasonography, no increased perfusion can be detected in the solid part (curved array). **(C)** Distribution pattern of contrast bubbles within the solid parts after 2 min. Strong enhancement with bizarre distribution within the irregularly configured solid mass (long arrow). **(D)** MRI of the pelvis shows the bizarre aspect of the solid mass with hyperintense cysts (black star) and irregularly configured hypointense stroma (black dashed arrow), (T2 TSE in paracoronal orientation).

A 10-year-old girl with abdominal pain and a large abdominal mass palpable for a fortnight was presented. The tumor marker alpha-fetoprotein (AFP) in serum was slightly elevated at 15 ng/ml. The other tumor markers and laboratory values were unremarkable. B-mode ultrasound and a multimodal MRI with DWI and contrast media visualized a cystic solid mass of a maximum of 15.5 cm depending on the left ovary. A contained irregularly configured solid portion without calcifications was ~7.0 cm in size and with vigorous perfusion on Doppler ultrasonography (Figure 3). Analysis of the dynamic CEUS examination in the solid part showed a TTP of 10.4 s and an RT of 5.8 s, and relative PE was 117% (Figures 1B,D). Purely visual examination revealed very vigorous and irregular flooding of the bubble in the solid part (Figure 3C). The findings in multimodal MRI with contrast media suspected an immature teratoma with additional peritoneal tumor seeding. Therefore, after determination from the interdisciplinary tumor board, an abdominal laparotomy was performed. During surgery, no healthy ovarian tissue could be visually differentiated. Therefore, adnexal extirpation was performed. Intraoperatively, there was no peritoneal tumor seeding. Histologically, the findings of an ovarian teratoma were confirmed. In addition to mature cell differentiation, there were larger regions with immature cells with neuroectodermally differentiated parts in the sense of an immature teratoma in stage G1 according to Gonzalez-Crussi. There was no infiltration into the capsule. The removal was performed in healthy tissue (R0).

## Discussion

Based on the two different pediatric cases with an ovarian mass presented here, it can be shown that dynamic CEUS with subsequent quantitative analysis is a new diagnostic method that appears to be able to better differentiate lesions. An ovarian mass in girls rarely occurs and differentiation of this lesion is sometimes not straightforward (2). In this regard, a baseline transabdominal b-mode ultrasound and a complementary multimodal MRI with DWI and contrast media can well identify the characteristic features of ovarian masses (6). Both imaging methods can reliably determine tumor size, and also reliably distinguish cystic and solid parts contained therein (4).

Of particular importance in all diagnostic procedures is that vascularization is assessed, as it correlates with the invasive potential of a lesion (15). Thus, a high degree of vascularization and associated higher malignancy of a lesion is characterized by a dense network of small vessels. Also, perfusion is an important functional parameter and describes the vitality of a tissue. Various physiological and pathological changes can alter the vascularization and perfusion of different tissues or tumors. Doppler ultrasonography can visualize the vascularization of a lesion well, but the method is not sensitive enough to visualize perfusion even in very small vessels (16).

In contrast dynamic CEUS can visualize vascularization and perfusion in real-time using intravascular microbubbles. The echogenicity of the bubbles in a tissue corresponds to the volume of blood contained therein (17). According to Mehta et al., CEUS imaging can accurately assess neovascularization and better illuminate vascular abnormalities (8). Besides, special software can be used to objectively quantify various perfusion parameters. Thus, dynamic CEUS can help to differentiate between benign and malignant ovarian tumors in women by the purely visual, i.e., qualitative, assessment of a lesion as well as by the subsequent quantitative analysis (18).

Studies in adult women have already demonstrated the value of dynamic contrast-enhanced ultrasound in malignant ovarian tumors (9, 10). Malignant ovarian tumors showed higher peak enhancement (PE) intensity, delayed washout, and overall more enhancement with chaotic patterns than a benign lesion. Ultimately, CEUS was more specific than native B-scan or Doppler ultrasonography in differentiating ovarian carcinomas (19). Yang et.al also demonstrated in his study that ovarian carcinomas had increased perfusion values and area under the curve (AUC) was a significant value for diagnosis. AUC had the highest diagnostic confidence with a sensitivity of 87.3% and specificity of 80.8% (20).

To our knowledge, except from a single case reports (11), the diagnostic possibility of dynamic CEUS has not been currently reported in girls with an ovarian mass.

For example, Madenci et al. showed that girls who had in imaging a simple cyst in the ovary never associated it with malignancy (5). Predominantly solid ovarian tumors were malignant in 44% of cases and solid-cystic mixed tumors with elevated tumor markers were malignant in 40% of cases. Without elevated tumor markers, 5% of cases were malignant if they were larger than 10 cm (5). Suspicious solid lesions in the ovary have a heterogeneous perfusion pattern on Doppler ultrasonography. In our experience and as well-demonstrated in the here described two cases, these heterogeneous patterns are much better visualized on CEUS than on Doppler ultrasonography.

Our case of an immature teratoma with potential malignant changes is in agreement with all these findings in adults and children. The tumor was very large, 16 cm in diameter. Dynamic CEUS and quantitative functional analysis showed significantly higher relative values for PE, RT, WiAUC, and WiWoAUC in the suspicious solid part compared to the first case of a mature teratoma. It must be remembered that the calculated absolute values of PE, WiAUC, and WiWaAUC are not well-comparable in children. Only TTP and RT can be evaluated as absolute values in seconds. We believe the reason for this is the dependence of the quantitative values on the amount of ultrasound contrast media applied (21). But the amount of contrast media varied sometimes significantly due to the different age and body weight of the examined girls. Yet the data of different ovarian masses is lacking in pediatric cases.

In our case, we assessed our decision to the operation management by the result of the MRI with contrast enhanced media and DWI. DynCEUS did not play a role in the treatment decision. The target for pediatric surgeons is always to perform an ovary sparing surgery (OSS) according to the modern surgery strategies (22). However, in the case presented here no healthy ovarian tissue could be visually differentiated during surgery. This was confirmed in the pathology, there was no normal healthy tissue to identify. Therefore, it was the only option to perform a unilateral tumor salpingo-oophorectomy.

We think that dynCEUS could be a helpful tool in differentiating unclear lesions on the ovary, including mature and immature teratomas. However, dynCEUS cannot be the only examination modality, it is always only one component in the overall imaging. In addition, a high level of experience in the use of contrast-enhanced ultrasound is required. Rather, dynamic CEUS could be quite helpful to increase the diagnostic confidence for the presence of immature teratoma. For this purpose, we assess the accumulation of the ultrasound contrast media in the ovary over time and the shape of the vascular architecture. Very bizarre vessels seem to be indicative of a more malignant lesion. Thus, in the second case of the girl with an immature teratoma showed a rather chaotic image during the contrast media inflow compared to the first case with a mature teratoma. This image corresponds to underlying vessels formed in malignant lesions by tumor-angioneogenesis. Such tumor vessels, in contrast to non-pathological vessels, have different characteristics that have an impact on perfusion. Our data show that a longer absolute and relative RT exists in the solid part of the immature teratoma compared to the mature teratoma. The contrast microbubbles in the immature teratoma take a longer time to reach maximal accumulation. The reduced flow velocity of contrast microbubbles in pathologic vessels may be due to the aging, corkscrew-like vessel shape, and branching of tumor endothelial cells across the vessel lumen (23). However, larger prospective studies are absolutely necessary to obtain more information and knowledge about this. Therefore, a prospective study with more patients would be helpful. This should be multicenter, as only a few comparable cases occur at one center within 1 year.

## Summary—Conclusion

These two cases teratoma demonstrate that dynamic contrast-enhanced ultrasound with quantitative analysis can help differentiate ovarian masses in children and adolescents. Dynamic CEUS meets the requirements for imaging that is easy and quick to perform. It can be a helpful additional tool in the differentiation of tumors. Thus, it can have a complementary contribution in the planning of a ovarian sparing surgery. However, further pediatric studies of quantitative CEUS are needed to characterize typical patterns of findings, receive more data and to improve pre-operative predictive accuracy in the evaluation of ovarian lesions.

## Data Availability Statement

The original contributions presented in the study are included in the article/supplementary material, further inquiries can be directed to the corresponding author.

## Ethics Statement

The studies involving human participants were reviewed and approved by Ethikkommission Universitätsklinikum Jena (2021-2102). Written informed consent to participate in this study was provided by the participants' legal guardian/next of kin.

## Author Contributions

All authors listed have made a substantial, direct and intellectual contribution to the work, and approved it for publication.

## Conflict of Interest

The authors declare that the research was conducted in the absence of any commercial or financial relationships that could be construed as a potential conflict of interest.

## Abbreviations

AFP: Alpha-fetoprotein
CEUS: contrast-enhanced ultrasound
DWI: diffusion weighted imaging
dynCEUS: dynamic contrast-enhanced ultrasound
FDA: Food and Drug Administration
MRI: magnetic resonance imaging
PE: peak enhancement
RT: rise time
ROI: region of interest
TTP: time to peak
US: ultrasound
WiAUC: wash-in area under the curve
WiWoAUC: wash-in and wash-out area under the curve.
